# Paraventricular Nucleus P2X7 Receptors Aggravate Acute Myocardial Infarction Injury *via* ROS-Induced Vasopressin-V1b Activation in Rats

**DOI:** 10.1007/s12264-021-00641-8

**Published:** 2021-02-23

**Authors:** Wenjing Cheng, Yinggang Sun, Qin Wu, Kokwin Ooi, Yi Feng, Chunmei Xia, Danian Zhu

**Affiliations:** 1 Department of Physiology and Pathophysiology, School of Basic Medical Sciences, Fudan University, Shanghai, 200032 China; 2 Department of Cardiovascular Diseases, Xinhua Hospital Affiliated to Shanghai Jiao Tong University School of Medicine, Shanghai, 200092 China; 3 Department of Medical Technology, Jiangsu Vocational College of Medicine, Yancheng, 224005 China; 4 Department of Integrative Medicine and Neurobiology, School of Basic Medical Sciences, Fudan University, Shanghai, 200032 China

**Keywords:** PVN, P2X7 receptor, Vasopressin, Reactive oxygen species, c-fos, Myocardial ischemia

## Abstract

The present study was designed to investigate the mechanisms by which P2X7 receptors (P2X7Rs) mediate the activation of vasopressinergic neurons thereby increasing sympathetic hyperactivity in the paraventricular nucleus (PVN) of the hypothalamus of rats with acute myocardial ischemia (AMI). The left anterior descending branch of the coronary artery was ligated to induce AMI in rats. The rats were pretreated with BBG (brilliant blue G, a P2X7R antagonist), nelivaptan (a vasopressin V1b receptor antagonist), or diphenyleneiodonium (DPI) [an nicotinamide adenine dinucleotide phosphate (NADPH) oxidase inhibitor]. Hemodynamic parameters of the heart were monitored. Myocardial injury and cardiomyocyte apoptosis were assessed. In the PVN of AMI rats, P2X7R mediated microglial activation, while reactive oxygen species (ROS) and NADPH oxidase 2 (NOX2) were higher than in the sham group. Intraperitoneal injection of BBG effectively reduced ROS production and vasopressin expression in the PVN of AMI rats. Moreover, both BBG and DPI pretreatment effectively reduced sympathetic hyperactivity and ameliorated AMI injury, as represented by reduced inflammation and apoptosis of cardiomyocytes. Furthermore, microinjection of nelivaptan into the PVN improved cardiac function and reduced the norepinephrine (AE) levels in AMI rats. Collectively, the results suggest that, within the PVN of AMI rats, P2X7R upregulation mediates microglial activation and the overproduction of ROS, which in turn activates vasopressinergic neuron-V1b receptors and sympathetic hyperactivity, hence aggravating myocardial injury in the AMI setting.

**Supplementary Information:**

The online version contains supplementary material available at 10.1007/s12264-021-00641-8.

## Introduction

Acute myocardial ischemia (AMI), is one of the main culprits behind heart failure and left ventricular dysfunction [1, 2]. Abundant studies have shown that one of the pathophysiological causes of both the occurrence and exaggeration of heart failure is increased sympathetic drive and/or parasympathetic withdrawal [3–5]. Under ischemic conditions, sympathetic activation is induced by the release of endogenous chemicals by the ischemic myocardium, which sensitize neurons and later induce and augment the cardiac sympathetic afferent reflex [6–11].

The paraventricular nucleus (PVN), an important integrative site for the control of cardiovascular activity, contains neurons that project to the intermediolateral cell column of the thoracolumbar spinal cord and the rostral ventrolateral medulla. The PVN also controls sympathetic nerve activity and blood pressure. It consists of a heterogeneous group of magnocellular and parvocellular neurons that are largely clustered into anatomically distinct divisions [12–15]. Magnocellular neurons project to the posterior pituitary and parvocellular neurons project to areas of the brainstem and spinal cord that control cardiac and sympathetic nerve activity [16]. Besides, evidence indicates that the PVN contributes to both acute and chronic osmotically-driven increases in sympathetic nerve activity. The PVN contains vasopressinergic (VPergic) neurons and a dense complement of vasopressin (VP) receptors. The neurohormone VP participates in the homeostatic control of arterial pressure and volume status *via* binding to its corresponding V1a, V1b. and V2 receptors [17]. Of note, the docking of vasopressin to its receptor is known to add more workload and stress to the AMI-damaged heart, primarily and directly *via* arterial vasoconstriction, excess water reabsorption, and heighten sympathetic output. In general, these neurohumoral reactions and sympathetic output can be perceived as a compensatory mechanism, but they can increase the ventricular afterload (which depresses stroke volume and the amount of blood delivered to cardiac tissue) and the preload (fluid retention ensues and causes pulmonary edema and pulmonary hypertension), contributing to ventricular dilation and failure in the long run. This is also supported by a study showing that unilateral microinjection of VP into the PVN significantly increases mean arterial pressure (MAP), heart rate (HR), and renal sympathetic nerve activity, peaking at 10 min in normal rats [18].

The pathophysiology of dysregulated sympathetic activity in exacerbating heart failure inspired us to investigate how this process is initiated, because it may serve as the rationale for therapeutic intervention. It is essential to consider the bidirectional effect of neuronal-glial signaling in the PVN, given its functional role in regulating neurohumoral physiology and sympathetic output. Numerous studies have confirmed the presence of P2X7R upregulation in microglia-derived neuroinflammation and illustrate the functional role of P2X7R overexpression in stimulating microglial activation and proliferation [19–21]. Some researches have shown that cardiovascular-related signals lead to increased microglial activation, in turn contributing to the exacerbated neurohumoral activation in heart failure. Microglia maintain normal neuronal physiology and homeostasis in the central nervous system (CNS) either in their resting or immuno-surveillant stage [22]. Our previous experiments showed that the increased P2X7R expression in microglia elicited by ATP is closely associated with the development of inflammatory responses and neuronal sensitization [23]. Microglia release a variety of cytokines, chemokines, reactive oxygen species (ROS), tumor necrosis factor α (TNF-α), and interleukin 6 (IL-6). It is therefore plausible that inflammatory molecules generated by activated microglia relay this information to areas of the PVN that, in turn, regulate neurohumoral physiology. ROS that are involved in microglial activation are thought to be generated primarily by NADPH oxidase (NOX) and ROS are involved in the immune response. Furthermore, ROS act as signaling molecules for maintaining homeostasis: upon interaction between ROS and signaling protein molecules, ROS can exert oxidative modification that changes the structure and function of the signaling protein, and in turn, regulating a variety of cellular processes *via* oxidative stress-responsive pathways [24]. Evidence shows that ROS-induced damage favors more ROS production, causing a positive feedback loop which accelerates the progression of many neurodegenerative diseases [25–27]. In the present study we intended to define how microglia might contribute to the activation of VPergic neurons *via* ROS signaling and thereby increase the sympathetic nerve activity in AMI.

## Materials and Methods

### Chemicals and Reagents

2,3,5-triphenyl tetrazolium chloride (TTC), BBG, DPI, dimethyl sulfoxide (DMSO), urethane, α-chloralose, and pontamine sky blue were produced by Sigma-Aldrich (St. Louis, MO, USA). Isoflurane was from RWD Life Science (Shenzhen, China). Nelivaptan (also known as SSR149415) was purchased from Reston, VA, USA.

### Animals and Ethical Approval

All animals were treated in accordance with the National Institutes of Health Guide for the Care and Use of Laboratory Animals. All experimental protocols were approved and performed according to the guidelines of the Animal Care and Use Committee of Fudan University. All surgical procedures were performed under anesthesia, and every effort was made to minimize suffering. Some reports have noted that sex hormones (especially estrogen) result in gender-influenced differences in cardiovascular regulation, so we used males to avoid this. Male Sprague-Dawley rats weighing 275–320 g were obtained from the Animal Center of Fudan University. They were housed under a 12-h light/dark cycle with free access to food and water; room temperature was maintained between 23 °C and 24 °C.

### Experimental Protocol

Two separate protocols were used as follows:

#### Protocol 1

Forty-eight rats that survived AMI surgery were randomly divided into Sham, AMI, and AMI + BBG groups. The BBG groups received the non-competitive P2X7R antagonist BBG for 5 days (25 mg/kg per day i.p.). The sham and AMI rats were treated with vehicle (saline i.p.) before surgery, and then were subject to sham ligation or ligation for 4 h [23].

#### Protocol 2

Forty-eight rats that survived AMI surgery were randomly placed into Sham, AMI, AMI + DPI (diphenyleneiodonium, a NOX inhibitor), and AMI + nelivaptan groups. The rats received PVN microinjection of DPI (100 μmol/0.1 μL) or nelivaptan (40 ng/0.1 μL) 15 min before 4 h AMI surgery. The dose of nelivaptan was chosen based on previous reports [18]. The same volume of corresponding solvent was used as vehicle control.

### Coronary Artery Ligation

The left anterior descending branch of the coronary artery (LAD) was ligated in rats to induce a model of AMI. Briefly, anesthetized rats were fixed in the supine position and body temperature was monitored and kept at 35 °C–36 °C with a temperature-controlled surgical table, intubated, and mechanically ventilated with a respirator (DHX-50, Chengdu Instrument Co, Chengdu, China) while the thorax was opened [28]. A left thoracotomy was then performed through the 4th intercostal space by cutting the pectoralis muscles transversely into the thoracic cage. And then the heart was exteriorized and the pericardium was removed. The LAD was located and ligated 2 mm–3 mm from the origin with a 6–0 silk suture for 4 h. The ligation was confirmed as successful when the anterior wall of the LV turned pale. Upon completion of ligation, the lungs were inflated to displace air and the thoracotomy site was closed in layers. After approximately 2 min–5 min ventilation with room air, the rats were gradually weaned from the ventilator once spontaneous respiration had resumed and then they were closely supervised until fully conscious. Sham ligation was used as a control, in which the same procedure was carried out except that the LAD was not occluded. The LAD ligation was successful and the AMI model was characterized by a lower MAP, lead II ST elevation, and pale infarction as shown in additional file 1 (Table S1 and Fig. S1).

### PVN Microinjection

Prior to surgery, each rat received an intraperitoneal injection 7 mL/kg of the anesthetic agent (140 g urethane, 7 g chloralose, and 7 g borax, mixed well and diluted in saline). After that, a motorized stereotaxic system (Neurostar, Tubingen, Germany) was used for cannula implantation and drug injection. The head of the rat was fixed on the stereotaxic apparatus; the coordinates for the PVN were set at 1.6 mm–1.9 mm caudal to bregma, 0.3 mm–0.5 mm lateral to the midline, and 7.7 mm–8.0 mm deep from the dorsal surface according to the atlas of Paxinos and Watson [29]. Briefly, after each rat was anesthetized, fixed, and had the scalp exposed a small hole was drilled; the rat was intubated with a polyethylene tube and spontaneously breathed room air. A 1-μL Hamilton syringe (7000 series, knurled hub) was used for injection at 50 nL/min and the syringe was withdrawn 10 min after each injection.

At the end of the experiments, microinjection sites into the PVN were marked by microinjection of 2% pontamine sky blue (0.1 μL). The rats were killed by decapitation, and then the brains were removed, fixed, sectioned, and stained with 1% neutral red to identify microinjection sites. The location of each study was identified and mapped on diagrams of the rat brain (Fig. S2) according to the atlas of Paxinos and Watson [29].

### Collection of Blood, Brain, and Heart Tissue Samples

Rats were decapitated while still under anesthesia to collect trunk blood and tissue samples. The blood samples were centrifuged at 3000 rpm for 15 min and then stored at −80°C until assay. Rats were euthanized and the brain was rapidly removed and frozen, blocked in the coronal plane, and cut into 500-μm slices on a cryostat. The PVN tissue was punched out according to the method of Palkovits and Brownstein [30]. Briefly, a punch biopsy was obtained from the right and left PVN and from right and left peripheral tissue (in the same section) using a 10-gage needle stub (ID: 1.0 mm). In addition, some rats was rapidly transcardially perfused with 0.9% normal saline followed by 4% neutral buffered paraformaldehyde (Thermo Fisher). Tissues were extracted, equilibrated in 30% sucrose, sectioned coronally on a cryostat (Leica Biosystems) at 25 μm, and rinsed in 0.1 mol/L phosphate-buffered saline. Besides, heart tissue was rapidly removed and rinsed in cold normal saline, which was adsorbed by filter paper. Plasma and tissue samples were stored at − 80 °C until assay.

### Evans Blue/TTC and Hematoxylin/Eosin (HE) Staining

The Evans blue/TTC method was used to visualize the infarcted area. Before a rat was euthanized, 0.5 mL of Evans blue dye (2%) was injected into the left ventricular cavity to measure the myocardial ischemic area at risk. The heart was excised and cut into 2-mm horizontal slices down the vertical plane. The slices were then stained with 1% TTC and subsequently fixed in formalin for 20 min. In addition, the excised heart was cut into 3 transverse sections, embedded in paraffin, and sectioned at 4 μm. After deparaffinization and dehydration, the sections were stained with HE and observed under an optical microscope (CH type, Olympus, Tokyo, Japan).

### TUNEL Assay

Apoptotic cells in myocardial tissue that occurred following AMI were identified by dUTP nick end-labeling (TUNEL) using an apoptosis detection kit (Beyotime, China), according to the manufacturer’s instructions. Briefly, paraffin sections were prepared as described above. Slides were incubated in TUNEL reaction mixture (50 μL) for 1 h at 37 °C in the dark and in a humidified atmosphere. For positive control, slides were incubated with 20 μg/mL DNase-free Proteinase K (Beyotime, China) for 10 min prior to incubation with the TUNEL reagent. After the TUNEL staining, these slides were covered with a glass coverslip applied with mounting medium and viewed under a Fluorview FV300 laser scanning confocal microscope. Immunoreactivity manifested as red fluorescence. TUNEL-positive cells were counted in six fields of five separate sections.

### Cardiac Magnetic Resonance Imaging (cMRI)

For cMRI, we infused drugs/vehicles into the PVN before 4 h AMI induction and acquired images using a Burke 7.0-Tesla horizontal-bore magnet controlled by a Unity Inova spectrometer (Palo Alto, CA, USA). Briefly, rats were anesthetized with 2%–4% isoflurane in an air-adjusted respiratory monitoring device. An echocardiogram -gated gradient echo sequence (fast low angle shot [FLASH]) was planned in the short-axis orientation. The cine images were acquired from 5 consecutive slices along the short cardiac axis at a slice thickness of 2.0 mm. Left ventricular (LV) end-diastolic volume, LV end-systolic volume, and LV mass (LVM) were determined by manual contouring of the endocardial and epicardial borders of the short-axis cine images using Segment software (Medviso AB, http://www.medviso.com), and LV wall volume and LVM were calculated by slice summation*.* Meanwhile*,* LV ejection fraction (LVEF) and LV cardiac output (CO) were also determined by Segment analysis.

#### Measuring Superoxide Anion Production in the PVN

Homogenates were prepared from the PVN samples. Total protein concentration was determined using a bicinchoninic acid protein assay kit (Pierce, Rockford, IL). Superoxide anion production was measured using the lucigenin chemiluminescence method (TD-20/20 Luminometer, Turner Designs). NADPH (100 μmol/L) and dark-adapted lucigenin (5 μmol/L) were added to 0.5-mL microcentrifuge tubes just before reading. Light emission was recorded over 10 min, and values are expressed as mean light units/min/mg protein.

#### ***In situ*** Detection of O^2−^ Production in the PVN

*In situ* superoxide anion levels in the PVN were determined by fluorescent-labeled dihydroethidium staining (DHE, Molecular Probes). Brain sections (20 μm) were incubated with 1 mmol/L DHE at 37°C for 10 min. Sections were imaged using a Nikon epifluorescence microscope.

#### Cardiac Structure and Function Evaluation

The changes of MAP, HR, and the left intraventricular pressure (LIVP) were monitored with a physiological signal acquisition system. Myocardial apoptosis was detected by the TUNEL method, and the mRNA of apoptotic (caspase-3 and caspase-9) and stress-related molecules (HSP27) were measured by RT-PCR. Cardiac function, including LV systolic pressure (LVSP), LV end-diastolic pressure (LVEDP), and maximum rate of rise/fall of LV pressure (±dp/dt_max_) were measured using a polygraph (Model SMUP-A, Department of Physiology and Pathophysiology, Shanghai Medical College of Fudan University), and cardiac tissue histology was examined, after AMI and drug treatment.

#### Measurement of NE Levels in Plasma

Serum samples were separated from plasma and stored at − 80 °C until assay for NE, the levels of which were measured by ELISA according to the manufacturer’s protocols.

#### Immunohistochemical and Immunofluorescent Staining

For immunohistochemical and immunofluorescent staining, rats were anesthetized with pentobarbital sodium (50 mg/kg, i.p.) and the ascending aorta was rapidly and thoroughly perfused with 200 mL 0.9% normal saline followed by 200 mL freshly-prepared 4% paraformaldehyde, The fixed brain was immersed in 20% sucrose until the it sank to the bottom, immersed in 30% sucrose at 4 °C overnight, and then coronal sections were cut at 25 μm on a cryostat microtome (CM1900; Leica Camera AG, Wetzlar, German). Paraffin sections were prepared for detecting Iba-1 in the PVN of AMI rats by immunohistochemical staining. In brief, immunodetection of Iba-1 was performed using anti-Iba-1 (1:100, Abcam, ab15690) and anti-OX42 (1:100, Abcam, ab216355) as the primary antibodies. Sections were incubated with the primary antibody overnight at 4°C after heat-mediated antigen retrieval, and incubated with horseradish peroxidase (HRP)-labeled anti-mouse antibody at 37°C for 120 min. Slides were then processed by a conventional procedure using 3,3N-diaminobenzidine tetrahydrochloride HRP color development kits. The images were captured under a light microscope (Leica FW 4000 Leica QWin system). Positive cell numbers were counted using ImageJ.

Moreover, double immunofluorescent staining of the PVN was used to detect co-localization of proteins. The sections were incubated with anti-vasopressin (1:100, Abcam, ab68669), anti-c-Fos (1:50, Santa Cruz, sc-8047), anti-NeuN (1:200, Abcam, ab104224), and anti-P2X7R (1:100, Abcam, ab109054) overnight at 4°C. The secondary antibodies consisted of goat anti-mouse conjugated with Alexa Fluor 488 and goat anti-rabbit conjugated with Alexa Fluor 594 (1:200, Life Technologies, UK). The sections were incubated for 2 h, and then cover-slipped with anti-fluorescence-quenching reagent. Immunofluorescence was detected using confocal microscopy and fluorescent intensity was measured using ImageJ software. These foci were then automatically segmented by thresholding, and pixel-by-pixel co-localization analysis of the segmented points from the two channels was assessed using the ImageJ plugin Just Another Colocalization Plugin (JACoP), which calculated Pearson coefficients, indicating the percentage of thresholded pixels in the green channel that was occupied by corresponding thresholded pixels in the red channel.

#### Western Blot Analysis

PVN tissue was homogenized in RIPA lysis buffer with protease and phosphatase inhibitors (Roche, Basel, Switzerland). The concentration of total proteins extracted was determined using a BCA assay kit. Equal quantities of each protein sample were separated by 10%–12% SDS-PAGE and transferred to a PVDF membrane. The primary antibodies used for Western blot analysis were anti-IL-1β (1:500, Abcam, ab9722), anti-NOX2 (1:2000, Abcam, ab129068), anti-P2X7R (1:200, Santa Cruz, sc-134224), and mouse anti-GAPDH (1:2000, Abcam, ab8245). This was followed by incubation with HRP-conjugated secondary antibody. The target proteins were detected using an ECL-Plus detection kit (Tiangen, Beijing, China), and scanned using Image Quant LAS 4000 (GE Healthcare Life Sciences, CT, USA). The images were quantified using the ImageJ densitometry system and expressed as the ratio to GAPDH protein.

#### Quantitative Real-time PCR Analysis

Total RNA from PVN was extracted by a RNAeasy kit (Qiagen, Germany) according to the manufacturer’s protocol. Extracted RNA was quantified by spectrophotometry and the optical density 260/280 nm ratio was determined. cDNA synthesis was performed using the ReverTra Ace qPCR RT Kit (Toyobo, Osaka, Japan), and RT-PCR was performed with SYBR-Green Realtime PCR Master Mix (Toyobo, Osaka, Japan) detected by the CFX Real-Time PCR System (Bio-Rad, Hercules, CA, USA). Relative levels of mRNA expression were normalized to GAPDH. The target genes and their primer sequences are listed in Table 1. The amplified cDNA was analyzed by agarose gel electrophoresis. After staining with ethidium bromide, gel images were captured under UV light.

**Table 1:** Target genes and their primer sequences.

Gene	Sense Primer (5′–3′)	Antisense Primer (5′–3′)
HSP27	GCAACTCAGCAGCGGTGTCT	TGTTCATCCTGCCTTTCTTCGT
Caspase-3	GAAAGCCGAAACTCTTCATCAT	ATGCCATATCATCGTCAGTTCC
Caspase-9	CCACTGCCTCATCATCAACAAC	GCCGTGACCATTTTCTTAGCAG
GAPDH	TTCCTACCCCCAATGTATCCG	CATGAGGTCCACCACCCTGTT

## Statistical Analysis

Data are expressed as the mean ± SEM. All analysis was performed using GraphPad Prism 6.0 software. When only two groups were compared, the paired or independent *t*-test was used as appropriate. When more than two groups were compared, generally one-way analysis of variance (ANOVA) followed by Bonferroni correction was applied, and an additional paired *t*-test was used when appropriate. The significance level was set at *P* < 0.05.

## Results

### AMI Induces the Upregulation of P2X7R, and Pretreatment with BBG, a P2X7R Inhibitor, Reduces Microglial Activation and Neuroinflammation in Rats

**Fig. 1:**
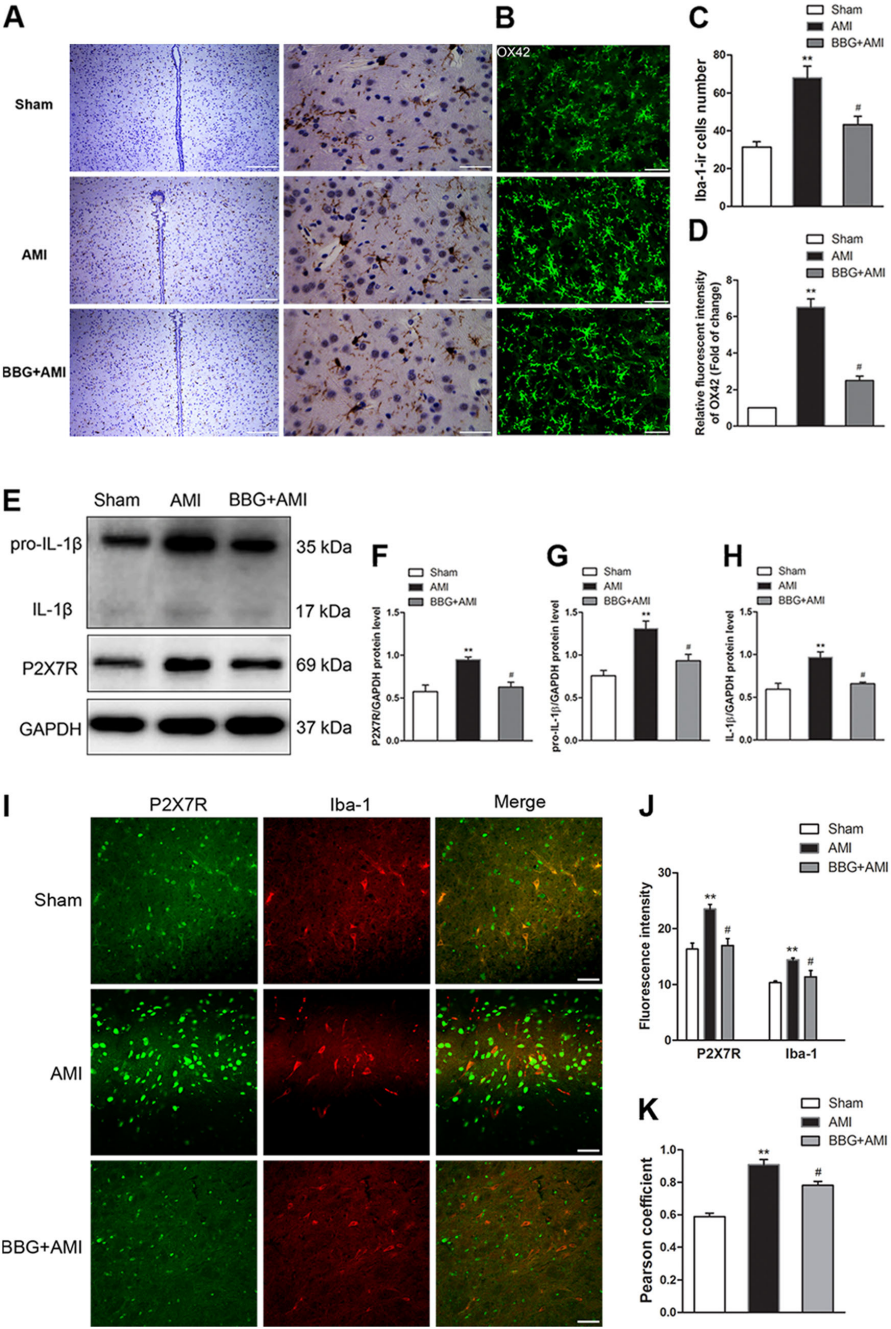
P2X7R expression and microglial activation in the PVN of AMI rats. **A** Representative image of immunohistochemistry of Iba-1-ir cells (brown) (scale bars, left 200 μm; right 50 μm). **B** Fluorescence images of microglia immunostained for anti-OX42 (cd11b/c) in the PVN from Sham, AMI, and BBG + AMI rats (scale bars, 50 μm). **C** Numbers of Iba-1-ir cells in the PVN. **D** Mean densitometry of OX42 (cd11b/c) immuno-positivity. **E** Representative immunoblot bands of P2X7R, pro-IL-1β, and IL-1β in the PVN in the different groups. **F**–**H** Optical density analysis of P2X7R (**F**)**,** pro-IL-1β (**G**) and IL-1β (**H**) immunoblotting bands. **I** Double immunofluorescence showing co-localization of P2X7R and Iba-1 analyzed by confocal fluorescence microscopy (scale bars, 20 μm). **J** Quantitative analysis of fluorescence intensity of P2X7R and Iba-1 staining in microglia from the groups. **K** Levels of co-localization of P2X7R and Iba-1 assessed using the Pearson coefficient. Data are expressed as the mean ± SEM. **P < 0.01 versus Sham group, ^#^*P* < 0.05 versus AMI group.

The LAD ligation was successfully performed in rats and the AMI model was characterized by lowered MAP, higher HR, lead II ST elevation, and pale infarction (Table S1 and Fig. S1). To demonstrate that activated P2X7R leads to the upregulation of activated microglia, we analyzed the relationship between the quantity of microglial marker (Iba-1) and the pro-inflammatory factor produced by activated microglia (protein expression of IL-1β and pro-IL-1β) in the PVN of AMI rats [31]. In this model, microglial activation was represented by accumulated Iba-1-ir cells and an intense OX-42 fluorescence signal (Fig. 1A, B). This result was correlated with the increased Iba-1-ir cell numbers and mean densitometry of OX42 positivity in quantitative studies (Fig. 1C, D, *n* = 3, *P* < 0.01). The immunoblotting bands of pro-IL-1β, IL-1β, and P2X7R were denser in the AMI group than in the sham group (Fig. 1E–H, *n* = 3, *P* < 0.01), while pretreatment with BBG, a P2X7R antagonist, reduced their expression (Fig. 1 A–H, *n* = 3, *P* < 0.05). P2X7R/Iba-1 co-localization showed that P2X7R was considerably expressed in microglia (Fig. 1I). Compared with AMI rats, those pretreated with BBG had significantly lower fluorescence intensity of Iba-1 positive cells and P2X7R immunopositive (ir) cells in the PVN (Fig. 1J, *n* = 3, *P* < 0.05). Pearson correlation coefficients also showed that BBG treatment attenuated the upregulation of the level of P2X7R/Iba-1 co-localization caused by AMI (Fig. 1K, *n* = 3, *P* < 0.05). Therefore, these results showed that pretreatment with BBG effectively inhibited P2X7R-mediated microglial activation and neuroinflammation in the PVN of AMI rats.

### AMI Induces the Upregulation of NOX2 Expression and ROS Production, and Pretreatment with BBG Reduces Oxidative Stress in the PVN of AMI Rats

**Fig. 2:**
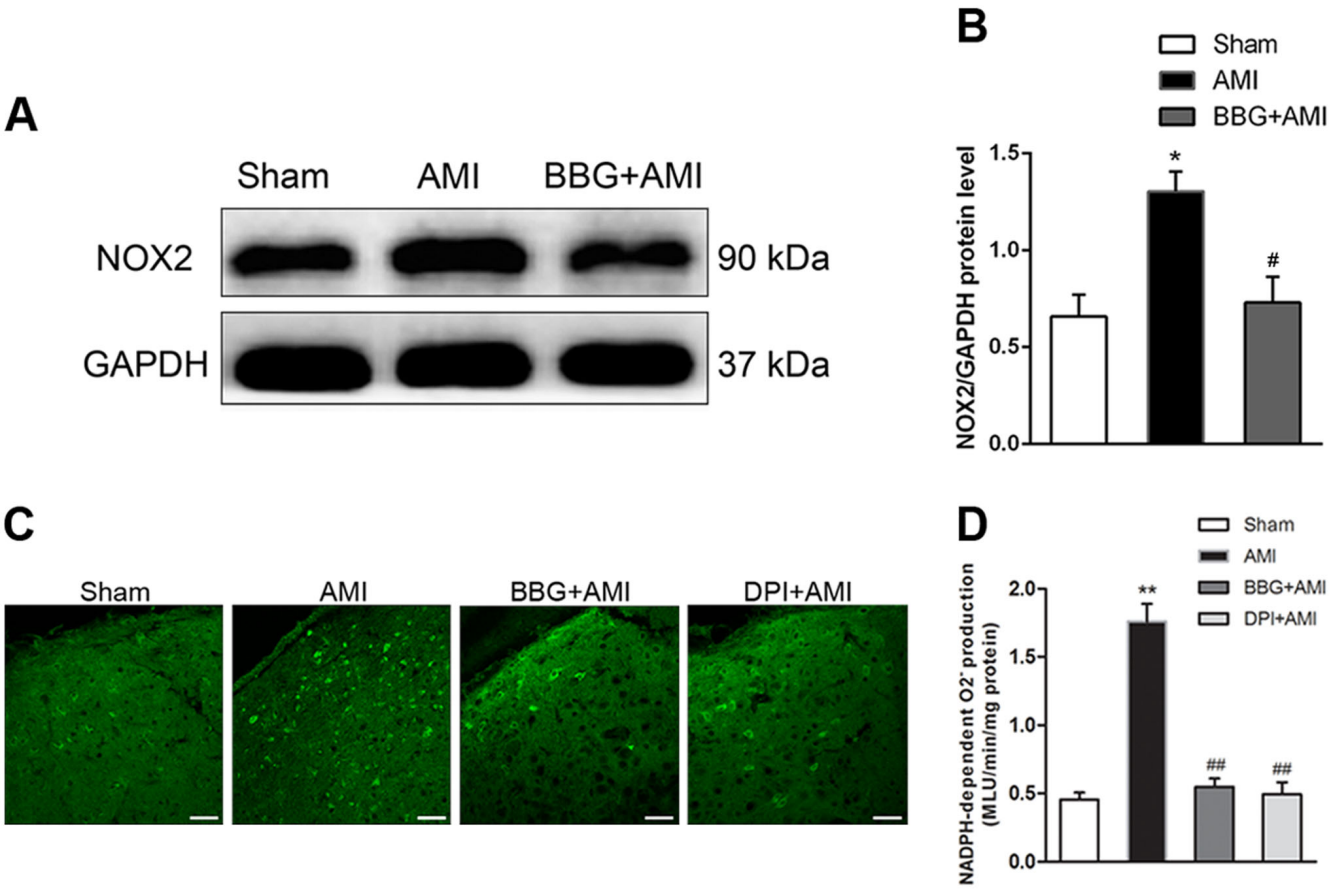
Effects of the P2X7R antagonist BBG on NOX2 expression and ROS production in the PVN of AMI rats. **A** Representative images of western blots showing protein expression of NOX2. **B** Histogram of protein levels relative to GAPDH levels. **C** Representative images showing the enhanced DHE fluorescence in the PVN of AMI compared with sham rats and those with other treatments (scale bars, 20 μm). **D** Mean NADPH-dependent O^2−^ production in the PVN of different groups. Data are presented as the mean ± SEM. **P < 0.01, *P < 0.05 versus Sham group, ^##^*P* < 0.01, ^#^*P* < 0.05 versus AMI group.

It has been reported that activation of NAD(P)H oxidative subunits (especially NOX2) is the main source of ROS production in the PVN, and this evokes overexpression of superoxide anion, consequently promoting sympathetic activity [32]. To investigate ROS accumulation in the PVN in the event of AMI, we used immunoblotting and densitometry to compare the levels of NOX2 in the sham *versus* AMI groups, and found that the level of NOX2 in AMI was approximately double the sham level (Fig. 2A, B, *n* = 3, *P* < 0.05). Besides, we utilized fluorescent probe assays to demonstrate ROS accumulation. In line with the previous results, quantitation of fluorescence imaging showed a significant increase in ROS fluorescent intensity in the AMI group relative to sham (Fig. 2C, D, *n* = 3, *P* < 0.01). Pretreatment with BBG reduced the NOX2 expression and ROS overproduction induced by AMI in the PVN (Fig. 2A–D, *n* = 3, *P* < 0.01 or *P* < 0.05).

### AMI Causes Increased Vasopressin Expression in Neurons, and Pretreatment with BBG or DPI, a NADPH Inhibitor, Reduces Vasopressinergic Neuronal Activation

**Fig. 3:**
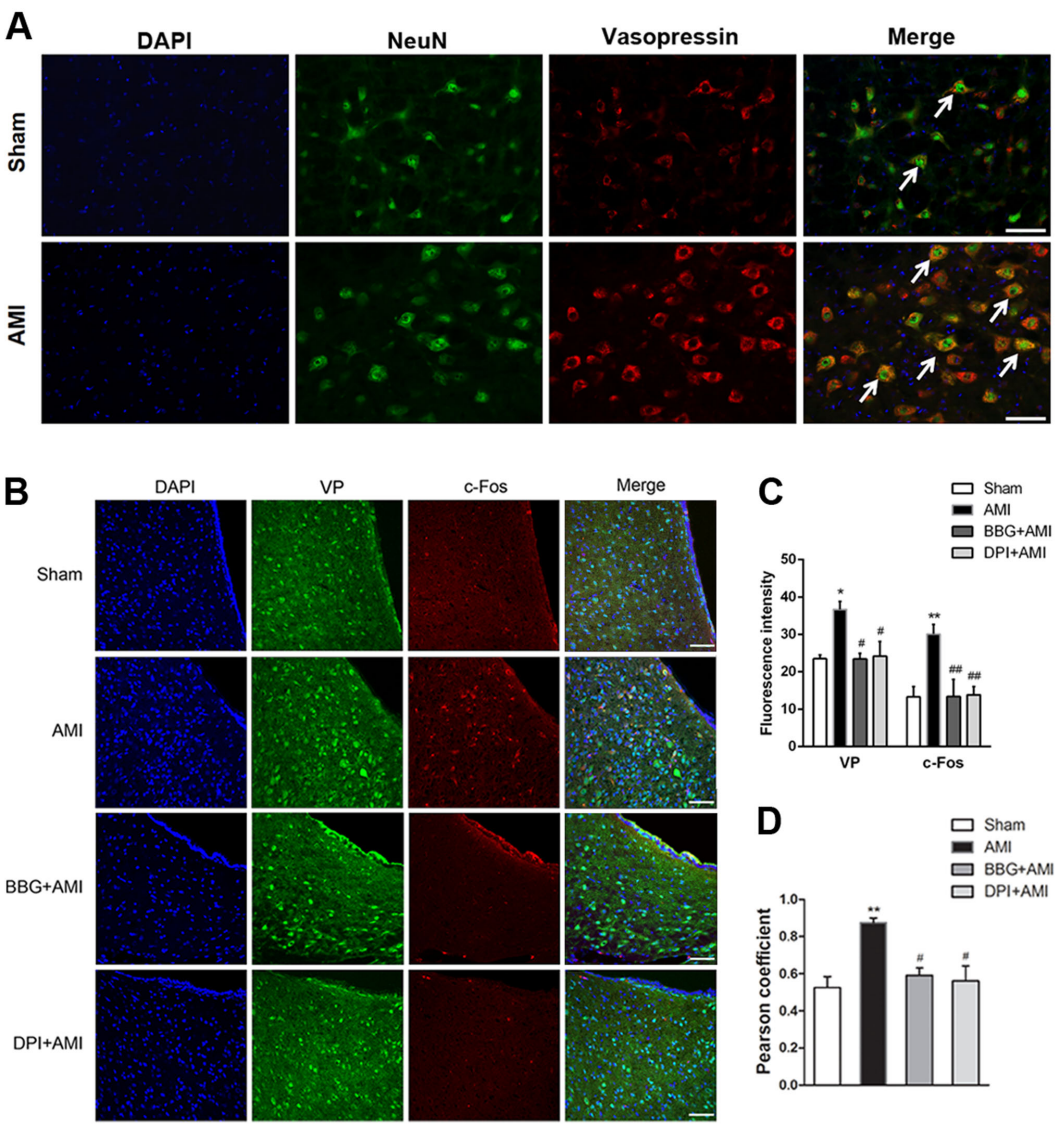
Pretreatment with BBG or an NADPH inhibitor (DPI) reduces VPergic cell activation. **A** Confocal microscopy images of double immunofluorescence showing co-localization of VP and NeuN (scale bars, 25 μm). **B** Confocal microscopy images of double immunofluorescence showing co-localization of c-Fos (red) and VP (green); nuclei stained with DAPI (blue) (scale bars, 20 μm). **C** Statistics of mean fluorescence intensity of c-Fos-ir and VP-ir in the PVN among groups. **D** Levels of co-localization of c-Fos and VP assessed using Pearson coefficients. Data are presented as the mean ± SEM. **P < 0.01, *P < 0.05 versus Sham group; ^##^*P* < 0.01, ^#^*P* < 0.05 versus AMI group. Ir, immunopositivity.

We continued to evaluate the role of VP as a neurotransmitter in regulating the cardiovascular autonomic nervous system. The double immunofluorescence-staining method was used to co-label VP and NeuN (a neuronal marker), which showed that VP was mainly expressed in neurons. VPergic cells were seen as red fluorescence, and NeuN as green. Compared to the sham group, the AMI group had more of VP-positive neurons in the PVN (Fig. 3A). To further evaluate whether the P2X7R-mediated ROS overproduction is responsible for triggering the activation of VPergic neurons following AMI, BBG was administered. VP-positive cells, c-Fos positive cells (representing immediate-early genes in the brain), and their co-localization (representing activated VPergic neurons) in the PVN were measured by double immunofluorescent staining (Fig. 3B). Compared with the sham group, the AMI group had more VP-positive cells and c-Fos immunopositive (ir) cells in the PVN (Fig. 3C, D, *n* = 3, *P* < 0.01 or *P* < 0.05). Pearson correlation coefficients showed that VP/c-Fos co-localization was higher in the AMI group than in the sham group, and after treatment with BBG or DPI, the correlation coefficients were reduced to values close to those of the sham group (Fig. 3B–D, *n* = 3, *P* < 0.05). Our results also demonstrated that the infusion of DPI or BBG into the PVN of sham rats did not elicit any of the above responses (data not shown). Collectively, these results indicated that the application of DPI or BBG to the PVN suppresses VPergic activity in the PVN of AMI rats.

### BBG Pretreatment Ameliorates Myocardial Ischemic Injury and Improves Cardiac Function in Rats with AMI

**Fig. 4:**
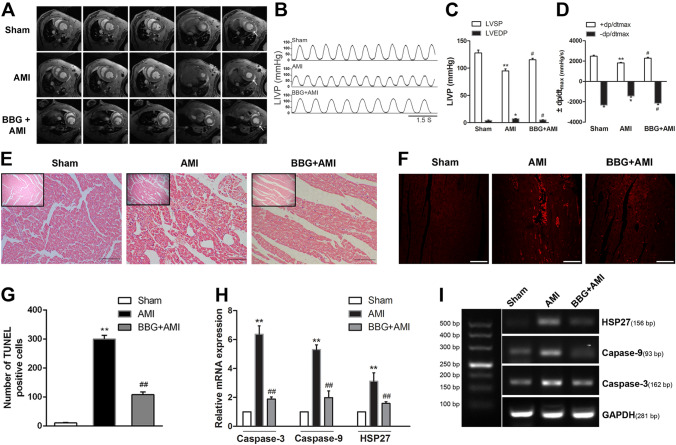
Cardiac function and heart histopathology. **A** Typical cMRI images from a cine study of the rat heart. Short-axis images spanning the base to apex are depicted in 5 slices, showing the radiological features in different groups of rats. Arrows indicate enhancement of the subendocardial late gadolinium. **B**, **C** LIVP recording and analysis before and after LAD ligation in rats. **D** Analysis of ±dp/dtmax. **E** HE staining of heart tissue showing histopathological changes post-AMI [scale bars, 200 μm (insert), 50 μm (below)]. **F**, **G** Representative images and analysis of apoptotic cardiomyocytes in myocardial sections using the TUNEL method (scale bars, 10 μm in **F**). **H** Expression of caspase-3, caspase-9, and HSP27 assessed by RT-PCR. **I** Agarose gel electrophoresis of the PCR products for caspase-3, caspase-9, HSP27, and GAPDH from individual samples (left lane: 500-bp molecular weight marker). Data are expressed as the mean ± SEM. **P < 0.01, *P < 0.05 versys Sham group; ^##^*P* < 0.01, ^#^*P* < 0.05 versus AMI group.

The rats received an intraperitoneal injection of BBG, a non-competitive P2X7R antagonist, before AMI surgery to evaluate whether P2X7R activation aggravates cardiac dysfunction in the AMI setting. In this context, we used cMRI as the reference standard for quantifying LV functions. Here, ischemic cardiomyopathy changes were characterized by the presence of subendocardial late gadolinium enhancement and these changes were recorded by cMRI in all AMI rats (Fig. 4A). In the AMI group, the greater the amount of myocardial tissue exhibiting delayed enhancement, the lower the chance of recovery of ventricular wall thickening that occurs after revascularization. In contrast, the BBG + AMI group showed less enhancement, and the greater area of viable myocardium was thus likely to improve following revascularization (Fig. 4A). Compared with the sham group, acute coronary artery occlusion indeed induced LV dysfunction at 4 h post-surgery (Table 2). BBG intervention ameliorated the dysfunctional LVEF caused by AMI, as shown by the higher LIVP recordings (Fig. 4B). In contrast to the BBG treatment groups, the LVSP (Fig. 4C, *n* = 5, *P* < 0.05), +dp/dtmax, and -dp/dtmax (Fig. 4D, *n* = 5, *P* < 0.05) in the AMI group were decreased and LVEDP (Fig. 4C, *n* = 5, *P* < 0.05) was increased, representing improved cardiac function in the BBG-treated group. Besides, the numbers of apoptotic cardiomyocytes (Fig. 4F, G, *n* = 3, *P* < 0.01), the mRNA of apoptotic molecules (caspas3 and caspase9), and heat shock protein HSP27 were decreased in the BBG-treated AMI heart, whereas they were markedly increased in the AMI group (Fig. 4H, I, *n* = 3, *P* < 0.01). Structural disarray and inflammatory cells were present in AMI myocardial tissue as shown in HE staining (Fig. 4E), while these pathological features were almost absent in the BBG-treated group.

**Table 2:** Changes in EDV, ESV, and EF after pretreatment with BBG in AMI rats.

Variable	Sham	AMI	BBG + AMI
LVM (mg)	562 ± 40.3	709.3 ± 35.3^*^	581.7 ± 27.4^#^
CO (ml/min)	163.6 ± 2.6	102.3 ± 6.0^**^	133.7 ± 4.1^#^
EF (%)	68.5 ± 2.5	51.50 ± 6.5^**^	63.0 ± 3.0^#^
EDV (mL)	0.73 ± 0.06	0.89 ± 0.05^*^	0.79 ± 0.02
ESV (mL)	0.41 ± 0.02	0.51 ± 0.01^**^	0.43 ± 0.03^##^

*LVM* left ventricular mass, *CO* cardiac output, *EF* ejection fraction, *EDV* end diastolic volume, *ESV* end systolic volume; Data are expressed as the mean ± SEM.

**P <  0.01, *P < 0.05 versus Sham group, ^##^*P* < 0.01, ^#^*P* < 0.05 versus AMI group, *n* = 3.

### BBG Treatment Reduces the Effect of AMI-Induced Hemodynamic Anomalies

**Fig. 5:**
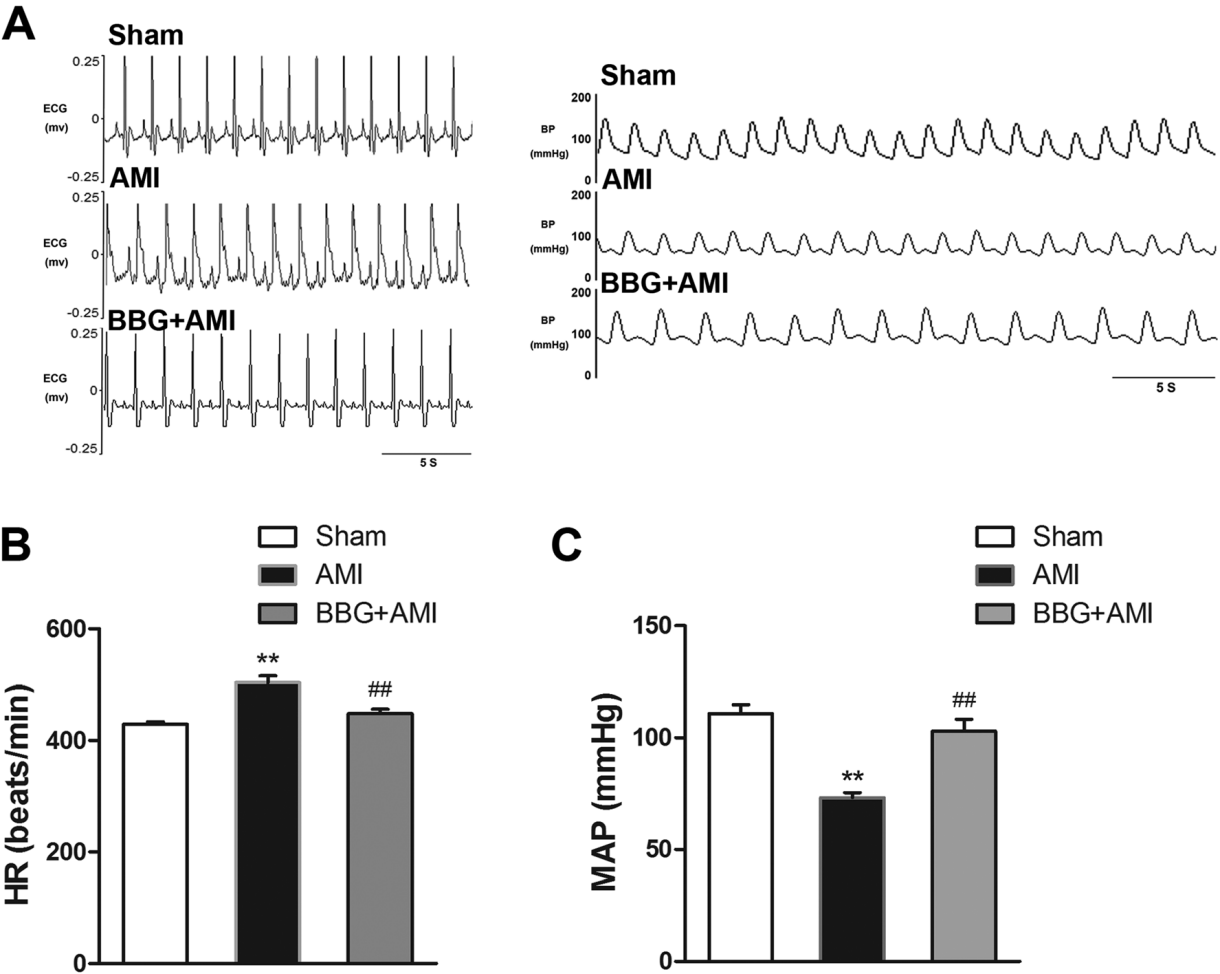
Protective effects of pretreatment with BBG on HR and MAP of AMI rats. **A** HR and blood pressure (BP) real-time recordings. **B**, **C** Statistics showing the protective effects of BBG on HR (**B**) and MAP (**C**) in AMI rats. Data are expressed as the mean ± SEM. **P < 0.01 versus Sham group, ^##^*P* < 0.01 versus AMI group.

Prolonged myocardial infarction is known to stun the myocardium, impair contractile function, and eventually cause myocyte death. Stunned and depressed contractile function (represented by decreased MAP) is detected by the cardiovascular regulatory center in the brain, and autoregulatory compensation then leads to heightened sympathetic output (represented by increased heart rate), which aggravates myocardial injury as time passes. We found that BBG significantly attenuated the increment of HR and decrement of MAP evoked by AMI (Fig. 5A–C, *n* = 6,* P* < 0.01), so it is plausible that a linkage exists between P2X7R activation and sympathetic overactivation responses.

### Pretreatment with BBG Reduces ROS Production, While Microinjection of DPI into the PVN is Protective Against Myocardial Injury *via* Decreasing NE Concentration in Serum

**Fig. 6:**
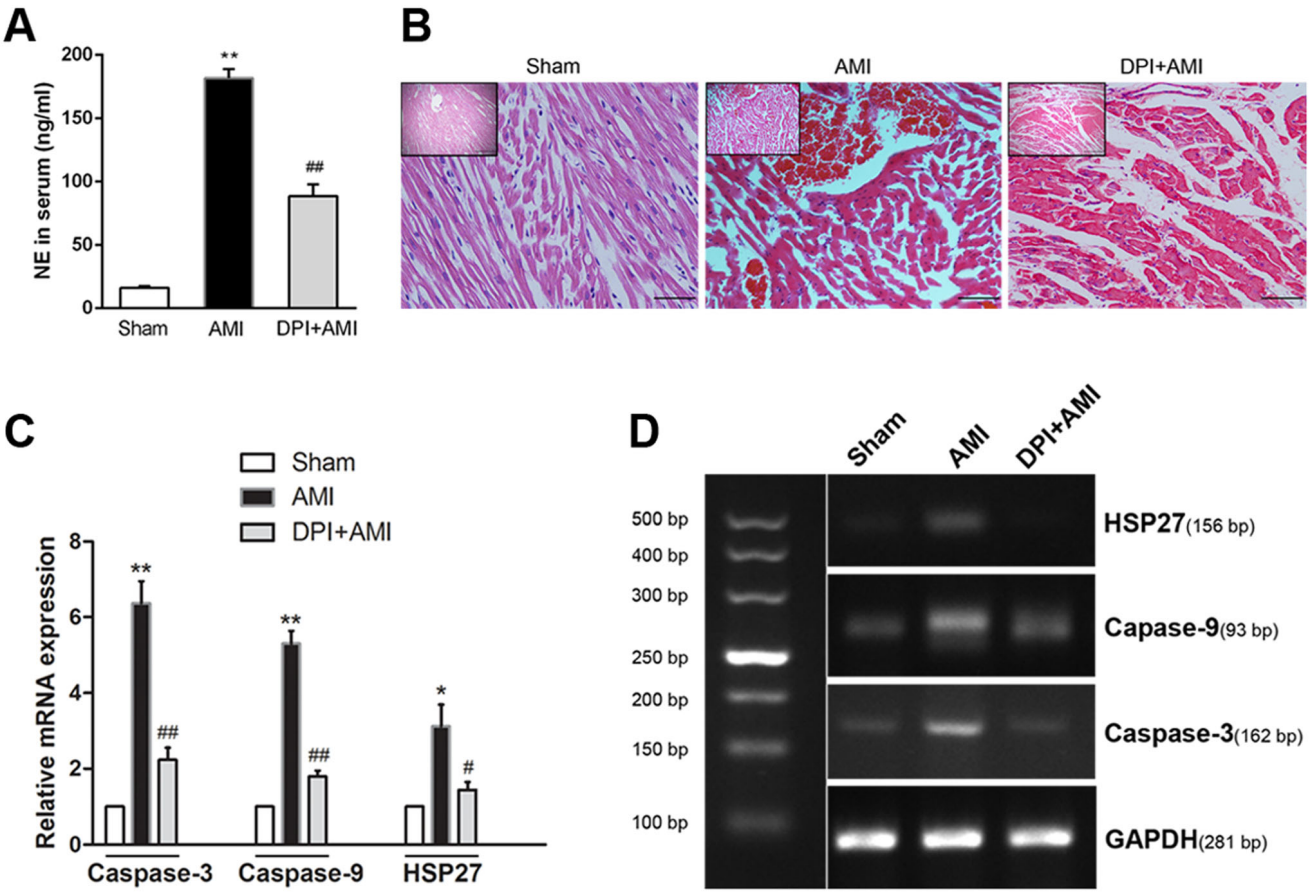
Protective effects of BBG and DPI on myocardial injury *via* increased sympathetic activity. **A** Serum levels of NE as measured by ELISA. **B** HE staining showing histopathological changes in heart tissue after AMI [(scale bars, 200 μm (insets), 50 μm (below)]. **C** Gene expression of caspase-3, caspase-9, and HSP27 mRNA by RT-PCR analysis. **D** Agarose gel electrophoresis of PCR amplicons for caspase-3, caspase-9, HSP27, and GAPDH from individual samples. Data are expressed as the mean ± SEM. **P < 0.01, *P < 0.05 versus Sham group; ^##^*P* < 0.01, ^#^*P* < 0.05 versus AMI group.

DHE fluorescent probe detection showed the total ROS in the PVN of AMI were significantly increased in AMI rats compared with the sham groups, while BBG or DPI pretreatment decreased the ROS production (Fig. 2C, D, *n* = 6, *P* < 0.01). Interestingly, both inhibitors showed a similar reduction in value in terms of lucigenin chemiluminescence from the peak value in the AMI group to near that of the sham group. To confirm the involvement of the ROS signal in the PVN that contributes to sympathetic hyperactivity in AMI, the rats were microinjected with DPI into PVN to monitor its effect on serum NE. The AMI group had an increased circulating serum NE concentration, while DPI reduced the level in AMI rats (Fig. 6A, *n* = 6, *P* < 0.01). Similarly, protective effects of DPI on myocardial injury were found *via* cardiac histopathology (Fig. 6B) and apoptotic gene mRNA measurement (Fig. 6C, D, *n* = 3, *P* < 0.01 or *P* < 0.05). DPI pretreatment reduced the level of the myocardial injury-related molecules HSP27 and apoptotic gene (caspase-3 and caspase-9) expression. And the myocardium exhibited less structural disarray and fewer inflammatory cells. These results implied that the protective effects of BBG on myocardial injury are bridged *via* reducing ROS production, thus attenuating sympathetic hyperactivity.

### Pretreatment with V1b Antagonist Restores Hemodynamic Function in AMI Rats

**Fig. 7:**
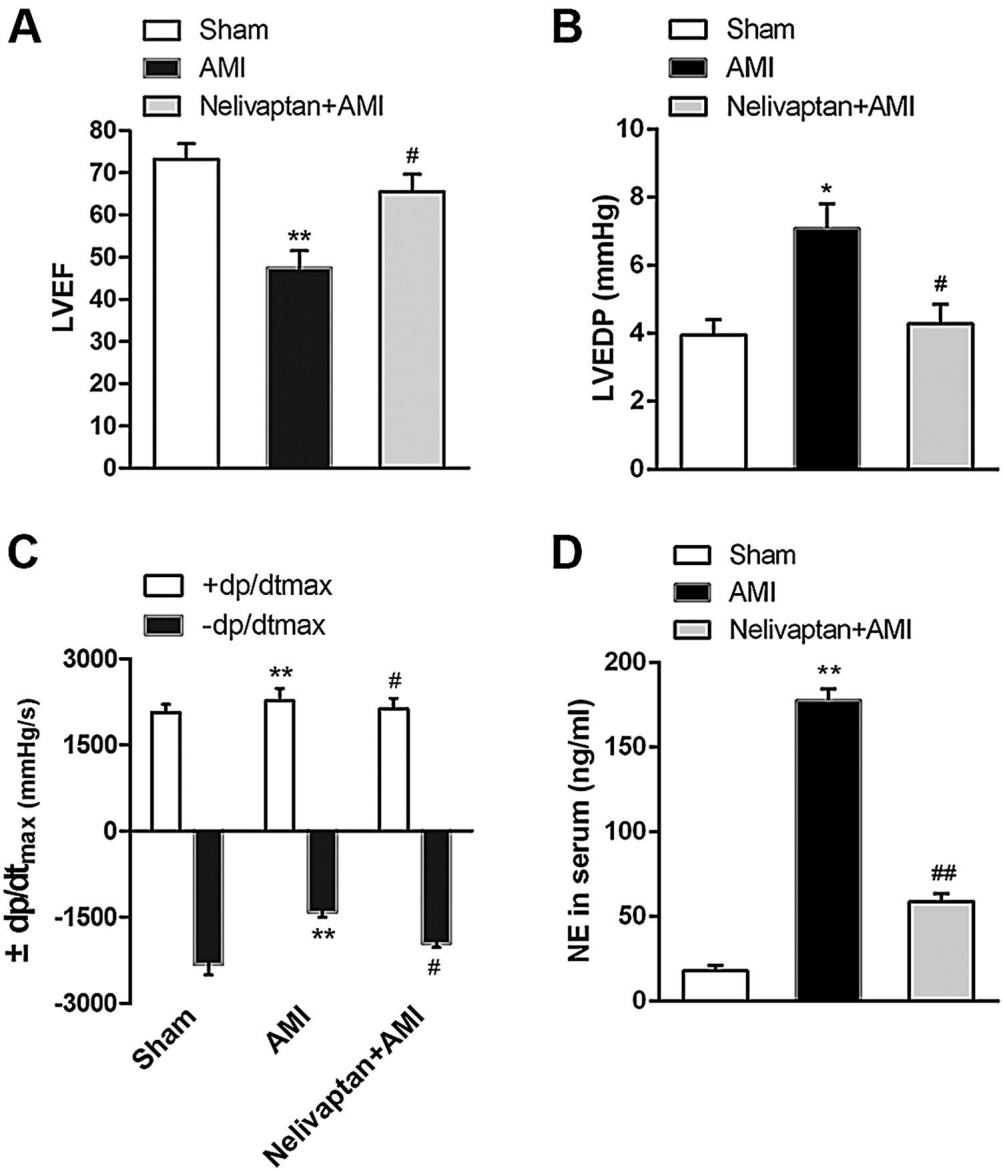
Inhibition of V1b receptors attenuates the hemodynamic dysfunction and reduces the NE level in AMI rats. **A** Analysis of change in LVEF in both groups. **B**, **C** Analysis of hemodynamic parameters LVEDP and dP/dtmax. **D** NE concentration in serum. Data are expressed as the mean ± SEM. **P < 0.01, *P < 0.05 versus Sham group, ^##^*P* < 0.01, ^#^*P* < 0.05 versus AMI group.

Compared with the AMI group, HR was significantly decreased while MAP was significantly increased in the nelivaptan pretreatment AMI group. Nelivaptan pretreatment ameliorated the dysfunctional LVEF (Fig. 7A, *n* = 3, *P* < 0.05) caused by AMI. In contrast to the nelivaptan treatment groups, LVEDP (Fig. 7B, *n* = 3, *P* < 0.05) was increased while +dp/dtmax /-dp/dtmax (Fig. 7C, *n* = 3, *P* < 0.01) was decreased in the AMI group. NE concentration in the serum of nelivaptan-treated rats was lower than in the AMI group (Fig. 7D, *n* = 3, *P* < 0.01). Microinjection of the VP receptor inhibitor-V1b antagonist nelivaptan into the PVN restored hemodynamic function of and NE level in AMI rats.

## Discussion

P2X7 receptors have a regulatory effect on cardiovascular activity [33]. Our study focused on the function and mechanism by which P2X7Rs control the sympathoexcitatory response using rats with AMI [23]. Occlusion of a major coronary artery in small rodents, followed by reperfusion or not, has proven to be a good model to assess the relevance of pathophysiological processes and drug effects in the setting of myocardial ischemia [34]. Besides, this model is currently used for pathophysiological studies and drug evaluation for myocardial infarction, left ventricular dysfunction, and heart failure [35, 36].

In AMI, both overactivation of the sympathetic nervous system and parasympathetic dysfunction occur [37]. During AMI, enhanced activity of the cardiac sympathetic afferent reflex (CSAR) transmits afferent input to and stimulates the cardiovascular center. The PVN is one of prominent cardiovascular regulatory centers, senses the afferent CSAR input, and reacts accordingly by upregulating VPergic activity and increasing sympathetic activity *via* direct neuronal projections to the peripheral sympathetic nervous system [38]. However, these actions add an extra workload to the AMI-damaged heart and aggravates myocardial ischemic injury in the long run [12]. Accumulating studies have shown that ATP acts as an agonist that activates P2 receptors, and modification of the P2 cation channel leads to depolarization of the target cell, subsequently triggering an inflammatory response and inducing the release of pro-inflammatory cytokines such as interleukin-1β. BBG is a selective P2X7R antagonist. BBG inhibition of P2X7R activation and abolishing the downstream reactions implicate P2X7R activation in mediating the responses (for example, increases in sympathetic activity and NE level in serum) in our experiments. However, none of our protocols showed that activation of P2X7R is directly stimulated by ATP [23]. We chose intraperitoneal administration of BBG based on following reasons: (1) BBG penetrates the blood-brain barrier [39]. (2) Although BBG also blocks P2X4Rs, it blocks P2X7Rs 1,000 times more than P2X4Rs. (3) The toxicity of BBG is low and it is safe, as it is also used in the food industry. It can be administered both orally or by intravenous injection [40–42].

To address whether improved sympathetic modulation ameliorates cardiac injury, we examined cardiac function (LVM, CO, EF, EDV, and ESV), histopathological changes, myocardial apoptotic molecular markers, and heat shock protein HSP27. Myocardial apoptotic markers were used because the apoptotic cell death is a significant contributor to myocardial damage in patients with AMI and is involved in the process of subsequent LV remodeling and the development of heart failure [43]. Consistent with previous results, BBG inhibition showed a promising outcome in terms of improved cardiac function (reduced LVM representing a reduction in compensatory hypertrophy, and increased CO, and EF, EDV, and ESV representing improved cardiac function), a significant reduction in apoptotic cardiomyocytes and apoptotic molecules, and last but not least, less myocardial structural disarray and fewer inflammatory cells than in the AMI group. These results reinforce the role of P2X7R activation in aggravating myocardial injury.

Our study showed the protective effect of BBG on cardiac function was achieved by inhibiting P2X7R activation in the PVN of AMI rats. Questions remain about how activated P2X7R is linked to activated microglia. Many reports have shown that the activation of PVN microglia increase the release of cytokines *via* P2X7R activation. For instance, the activation of P2X7R induces Ca^2+^ influx and K^+^ efflux, leading to the activation of IL-1β by the formation of converting enzyme and promoting the production and release of IL-1β. Furthermore, neuroinflammation contributes to sympathetic nerve excitation [44, 45], which is supported by evidence that the microinjection of TNF-α and IL-1β into the rat PVN results in the enhancement of peripheral sympathetic nerve excitation [46–48]. In this paper, we found that the AMI group showed a significant increase in microglial activation, whereas BBG treatment reversed this effect. Moreover, P2X7R/Iba-1 double-positive immunoreactivity was increased in the AMI group and decreased in the BBG group. This strengthens the correlational relationship between P2X7R activation and the upregulation of activated microglia. However, a direct causal relationship requires further evaluation; P2X7R might be upregulated in other cell types other than microglia alone.

Since P2X7R overactivation in the PVN is regarded as the culprit behind sympathetic hyperactivity and the aggravation of cardiac dysfunction, we continued to study the mechanism of how activated microglia produce and relay signals, and act on VPergic neurons. It has been reported that, in the neuroinflammatory state, an increase in pro-inflammatory cytokines and activation of NF-кB in the PVN promote ROS production, which relays the signal to effector cells *via* the oxidative-stress response pathway [49]. Numerous studies indicate that ROS in the PVN are primarily produced by the activated microglia-derived NOX2 protein, which can be explained by the role of NOX2 in mediating the process of killing pathogens, neuroinflammation, and microglia-induced neurotoxicity. Although the presence of NOX2 and other NOX isoforms has been described in neurons and astrocytes, there is no detailed physiological knowledge concerning the expression of NOX isoforms. Convincing evidence also shows that the expression of NOX in neurons or astrocytes is not constitutive, but induced by neuroinflammation in a pathological state such as Alzheimer's disease and stroke [50]. We found that BBG blocked the upregulation of NOX2 and ROS production, which strengthened the P2X7R-mediated ROS production under AMI. We also found that microinjection of the NADPH oxidase inhibitor DPI into the PVN decreased the plasma levels of NE in AMI and protected the heart from ischemic injury, which suggested that ROS might mediate the effect of P2X7R on sympathetic hyperactivity.

VP is a hormone that is responsible for both osmotic and cardiovascular homeostasis. Goldsmith *et al.* demonstrated that VP adversely affects myocardial function *via* peripheral vasoconstriction, ventricular remodeling, and water retention [51]. Based on this premise, we first needed to confirm the elevation of VPergic neural activity in the AMI setting. Moreover, it is noteworthy that c-Fos expression in neurons provides a useful marker that indicates activated neurons and neural activity in the CNS in response to peripheral stimulation [52, 53]. We found that the number of co-localized c-Fos positive and VPergic neurons was significantly decreased *via* NADPH oxidase inhibition.

To identify the mechanism by which the activation of VPergic neurons aggravates myocardial injury in AMI rats, we studied the action of VP upon docking with its V1b receptor. Czarzasta *et al.* demonstrated that heart failure and a high-fat diet cause significant changes in the expression of APJR, V1aR, and V1bR, which may have an important influence on the cardiovascular system and metabolism [54]. Consistent with a previous study [18], we confirmed that microinjection of the V1b antagonist nelivaptan into the PVN attenuated the NE level and hemodynamic dysfunction, which was represented by a significant improvement in LVEF and dP/dt-max and a reduction in LVEDP in AMI rats.

## Conclusions

**Fig. 8:**
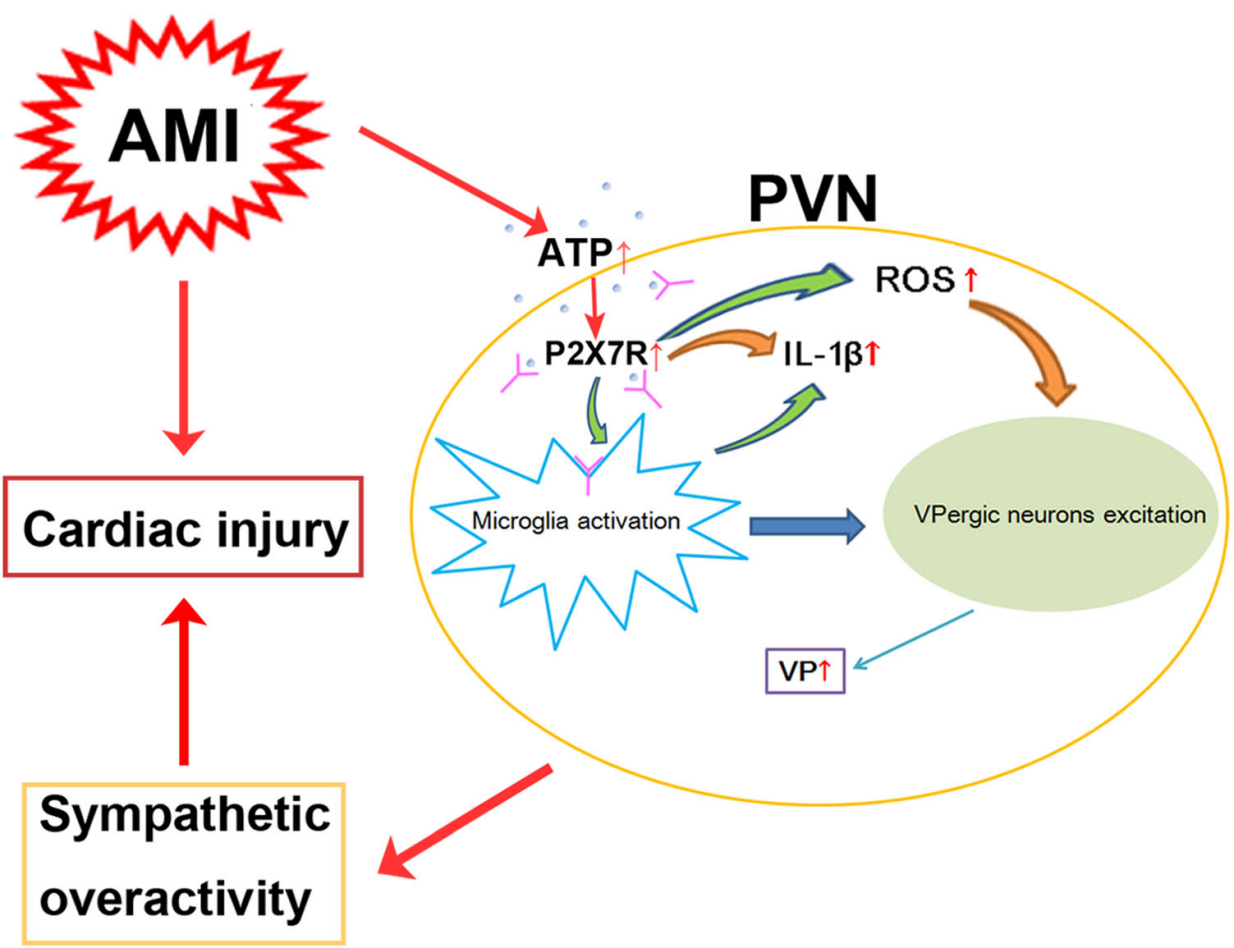
Schematic displaying that PVN P2X7R activation aggravates acute myocardial injury *via* ROS-induced vasopressin-V1b activation in AMI rats.

Following AMI, increased levels of ATP and/or IL-1β in the PVN drive P2X7R-induced microglial overactivation and ROS overproduction, which mediate the excitation of VPergic neurons, thereby causing sympathetic hyperactivity and aggravating cardiac dysfunction (Fig. 8).

## Limitations

There were several technical limitations in this study. First, the cellular localization of P2X7R in the CNS remains controversial. Therefore, further experiments are needed to define the P2X7 expression in different cell types. Second, the interactions among cell types noted above in the regulation of cardiac function need to be clarified under the setting of cardiovascular disorders. Last, we found it difficult to exclude the possibility that the ATP-induced microglial activation may be dependent on other similar adenosine receptors in the P2 receptor family (such as P2Y12R).

## Supplementary Information

Below is the link to the electronic supplementary material.

Supplementary file1 (PDF 569 KB)

## References

[1] Adabag AS, Therneau TM, Gersh BJ, Weston SA, Roger VL (2008). Sudden death after myocardial infarction. JAMA.

[2] Lin SK, Liu JM, Chang YH, Ting YT, Pang ST, Hsu RJ (2017). Increased risk of endotracheal intubation and heart failure following acute myocardial infarction in patients with urolithiasis: a nationwide population-based study. Ther Clin Risk Manag.

[3] Ramchandra R, Barrett CJ (2015). Regulation of the renal sympathetic nerves in heart failure. Front Physiol.

[4] Grippo AJ (2009). Mechanisms underlying altered mood and cardiovascular dysfunction: the value of neurobiological and behavioral research with animal models. Neurosci Biobehav Rev.

[5] Grippo AJ, Johnson AK (2009). Stress, depression and cardiovascular dysregulation: a review of neurobiological mechanisms and the integration of research from preclinical disease models. Stress.

[6] Wang WZ, Gao L, Wang HJ, Zucker IH, Wang W (2008). Interaction between cardiac sympathetic afferent reflex and chemoreflex is mediated by the NTS AT1 receptors in heart failure. Am J Physiol Heart Circ Physiol.

[7] Chen WW, Xiong XQ, Chen Q, Li YH, Kang YM, Zhu GQ (2015). Cardiac sympathetic afferent reflex and its implications for sympathetic activation in chronic heart failure and hypertension. Acta Physiol (Oxf).

[8] Grassi G, Quarti-Trevano F, Esler MD. Sympathetic activation in congestive heart failure: an updated overview. Heart Fail Rev 2019. 10.1007/s10741-019-09901-2 31832833

[9] Grassi G, Seravalle G, Mancia G (2015). Sympathetic activation in cardiovascular disease: evidence, clinical impact and therapeutic implications. Eur J Clin Invest.

[10] Barretto AC, Santos AC, Munhoz R, Rondon MU, Franco FG, Trombetta IC (2009). Increased muscle sympathetic nerve activity predicts mortality in heart failure patients. Int J Cardiol.

[11] Cheng ZJ, Wang R, Chen QH (2019). Autonomic regulation of the cardiovascular system: diseases, treatments, and novel approaches. Neurosci Bull.

[12] Kang YM, Yang Q, Yu XJ, Qi J, Zhang Y, Li HB (2014). Hypothalamic paraventricular nucleus activation contributes to neurohumoral excitation in rats with heart failure. Regen Med Res.

[13] Pyner S (2014). The paraventricular nucleus and heart failure. Exp Physiol.

[14] Ferguson AV, Latchford KJ, Samson WK (2008). The paraventricular nucleus of the hypothalamus-a potential target for integrative treatment of autonomic dysfunction. Expert Opin Ther Targets.

[15] Eliava M, Melchior M, Knobloch-Bollmann HS, Wahis J, Da SGM, Tang Y (2016). A new population of parvocellular oxytocin neurons controlling magnocellular neuron activity and inflammatory pain processing. Neuron.

[16] Pyner S, Coote JH (1999). Identification of an efferent projection from the paraventricular nucleus of the hypothalamus terminating close to spinally projecting rostral ventrolateral medullary neurons. Neuroscience.

[17] Thibonnier M, Coles P, Thibonnier A, Shoham M (2002). Molecular pharmacology and modeling of vasopressin receptors. Prog Brain Res.

[18] El-Werfali W, Toomasian C, Maliszewska-Scislo M, Li C, Rossi NF (2015). Haemodynamic and renal sympathetic responses to V1b vasopressin receptor activation within the paraventricular nucleus. Exp Physiol.

[19] Deuchars SA, Atkinson L, Brooke RE, Musa H, Milligan CJ, Batten TF (2001). Neuronal P2X7 receptors are targeted to presynaptic terminals in the central and peripheral nervous systems. J Neurosci.

[20] Sperlagh B, Kofalvi A, Deuchars J, Atkinson L, Milligan CJ, Buckley NJ (2002). Involvement of P2X7 receptors in the regulation of neurotransmitter release in the rat hippocampus. J Neurochem.

[21] Monif M, Reid CA, Powell KL, Smart ML, Williams DA (2009). The P2X7 receptor drives microglial activation and proliferation: a trophic role for P2X7R pore. J Neurosci.

[22] Hu L, Zhang S, Ooi K, Wu X, Wu J, Cai J (2020). Microglia-derived NLRP3 activation mediates the pressor effect of prorenin in the rostral ventrolateral medulla of stress-induced hypertensive rats. Neurosci Bull.

[23] Du D, Jiang M, Liu M, Wang J, Xia C, Guan R (2015). Microglial P2X(7) receptor in the hypothalamic paraventricular nuclei contributes to sympathoexcitatory responses in acute myocardial infarction rat. Neurosci Lett.

[24] Ray PD, Huang BW, Tsuji Y (2012). Reactive oxygen species (ROS) homeostasis and redox regulation in cellular signaling. Cell Signal.

[25] Szeto HH (2006). Mitochondria-targeted peptide antioxidants: novel neuroprotective agents. AAPS J.

[26] Andersen JK (2004). Oxidative stress in neurodegeneration: cause or consequence?. Nat Med.

[27] Di Carlo M, Giacomazza D, Picone P, Nuzzo D, San BP (2012). Are oxidative stress and mitochondrial dysfunction the key players in the neurodegenerative diseases?. Free Radic Res.

[28] Zeng X, He H, Yang J, Yang X, Wu L, Yu J, *et al*. Temporal effect of Guanxin No. 2 on cardiac function, blood viscosity and angiogenesis in rats after long-term occlusion of the left anterior descending coronary artery. *J Ethnopharmacol* 2008, 118: 485–494. 10.1016/j.jep.2008.05.017 18579111

[29] Paxinos G, Watson CR, Emson PC (1980). AChE-stained horizontal sections of the rat brain in stereotaxic coordinates. J Neurosci Methods.

[30] Palkovits M, Brownstein M. Brain microdissection techniques. In: Cuello A. E., editor. *Brain Microdissection Techniques* 1983, Chichester: John Wiley & Sons.

[31] Janks L, Sharma C, Egan TM (2018). A central role for P2X7 receptors in human microglia. J Neuroinflammation.

[32] Kang Y, Ding L, Dai H, Wang F, Zhou H, Gao Q, *et al*. Intermedin in paraventricular nucleus attenuates Ang II-induced sympathoexcitation through the inhibition of NADPH oxidase-dependent ROS generation in obese rats with hypertension. *Int J Mol Sci* 2019, 20. 10.3390/ijms20174217 PMC6747263 31466304

[33] Burnstock G (2017). Purinergic signaling in the cardiovascular system. Circ Res.

[34] Chimenti S, Carlo E, Masson S, Bai A, Latini R (2004). Myocardial infarction: animal models. Methods Mol Med.

[35] Rivard AL, Simura KJ, Mohammed S, Magembe AJ, Pearson HM, Hallman MR (2006). Rat intubation and ventilation for surgical research. J Invest Surg.

[36] Wu Y, Yin X, Wijaya C, Huang MH, McConnell BK. Acute myocardial infarction in rats. *J Vis Exp* 2011. 10.3791/2464 PMC3197402 21372786

[37] Wu P, Vaseghi M (2020). The autonomic nervous system and ventricular arrhythmias in myocardial infarction and heart failure. Pacing Clin Electrophysiol.

[38] Dampney RA, Michelini LC, Li DP, Pan HL (2018). Regulation of sympathetic vasomotor activity by the hypothalamic paraventricular nucleus in normotensive and hypertensive states. Am J Physiol Heart Circ Physiol.

[39] Donnelly-Roberts DL, Jarvis MF (2007). Discovery of P2X7 receptor-selective antagonists offers new insights into P2X7 receptor function and indicates a role in chronic pain states. Br J Pharmacol.

[40] Gum RJ, Wakefield B, Jarvis MF (2012). P2X receptor antagonists for pain management: examination of binding and physicochemical properties. Purinergic Signal.

[41] Jiang LH, Mackenzie AB, North RA, Surprenant A (2000). Brilliant blue G selectively blocks ATP-gated rat P2X(7) receptors. Mol Pharmacol.

[42] Metzger MW, Walser SM, Aprile-Garcia F, Dedic N, Chen A, Holsboer F (2017). Genetically dissecting P2rx7 expression within the central nervous system using conditional humanized mice. Purinergic Signal.

[43] Bennett MR (2002). Apoptosis in the cardiovascular system. Heart.

[44] Du D, Hu L, Wu J, Wu Q, Cheng W, Guo Y (2017). Neuroinflammation contributes to autophagy flux blockage in the neurons of rostral ventrolateral medulla in stress-induced hypertension rats. J Neuroinflammation.

[45] Zhang S, Hu L, Jiang J, Li H, Wu Q, Ooi K (2020). HMGB1/RAGE axis mediates stress-induced RVLM neuroinflammation in mice *via* impairing mitophagy flux in microglia. J Neuroinflammation.

[46] Wei SG, Yu Y, Zhang ZH, Felder RB (2015). Proinflammatory cytokines upregulate sympathoexcitatory mechanisms in the subfornical organ of the rat. Hypertension.

[47] Sasaki Y, Ohsawa K, Kanazawa H, Kohsaka S, Imai Y (2001). Iba1 is an actin-cross-linking protein in macrophages/microglia. Biochem Biophys Res Commun.

[48] Ito D, Imai Y, Ohsawa K, Nakajima K, Fukuuchi Y, Kohsaka S (1998). Microglia-specific localisation of a novel calcium binding protein, Iba1. Brain Res Mol Brain Res.

[49] Kang YM, Gao F, Li HH, Cardinale JP, Elks C, Zang WJ (2011). NF-kappaB in the paraventricular nucleus modulates neurotransmitters and contributes to sympathoexcitation in heart failure. Basic Res Cardiol.

[50] Rojo AI, McBean G, Cindric M, Egea J, Lopez MG, Rada P (2014). Redox control of microglial function: molecular mechanisms and functional significance. Antioxid Redox Signal.

[51] Goldsmith SR, Gheorghiade M (2005). Vasopressin antagonism in heart failure. J Am Coll Cardiol.

[52] Roy RK, Augustine RA, Brown CH, Schwenke DO (2018). Activation of oxytocin neurons in the paraventricular nucleus drives cardiac sympathetic nerve activation following myocardial infarction in rats. Commun Biol.

[53] Patel KP, Zhang K, Kenney MJ, Weiss M, Mayhan WG (2000). Neuronal expression of Fos protein in the hypothalamus of rats with heart failure. Brain Res.

[54] Czarzasta K, Wojno O, Zera T, Puchalska L, Dobruch J, Cudnoch-Jedrzejewska A (2019). The influence of post-infarct heart failure and high fat diet on the expression of apelin APJ and vasopressin V1a and V1b receptors. Neuropeptides.

