# Supplementary material for: Paraventricular Nucleus P2X7 Receptors Aggravate Acute Myocardial Infarction Injury via ROS-Induced Vasopressin-V1b Activation in Rats
Source: Neurosci Bull. 2021 Feb 23;37(5):641–56. doi: 10.1007/s12264-021-00641-8 (PMC8099953; doi:10.1007/s12264-021-00641-8)
Supplement: Supplementary file 1 — Supplementary file1 (PDF 569 KB) [file 12264_2021_641_MOESM1_ESM.pdf]

Supplementary Materials

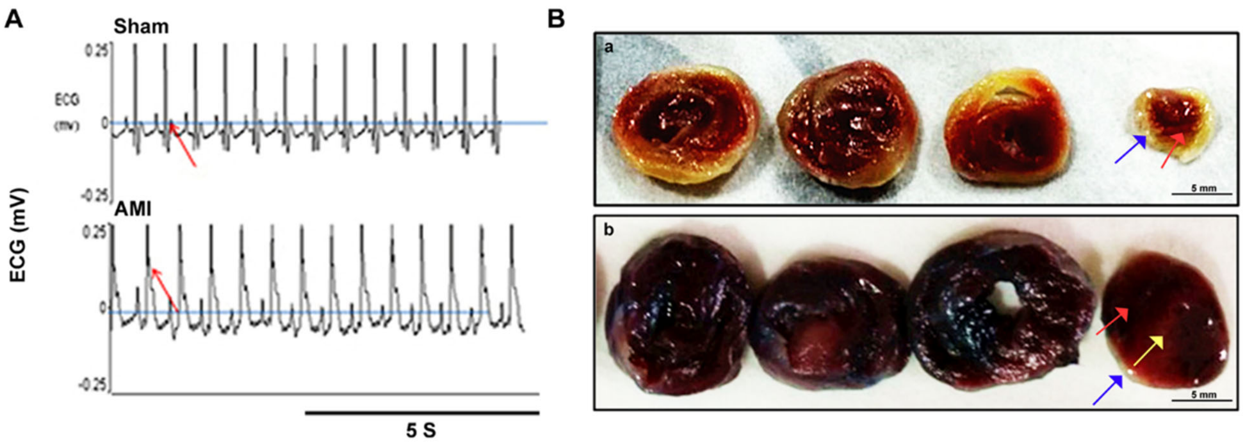

Fig S1 Assessment of acute myocardial ischemia by LAD ligation in rats. **A** ECG changes at lead II in the Sham and AMI groups. The follow-up ECG after AMI shows ST segment elevation at lead II lasting >30 min. **B** TTC staining (**a**) showing that the viable areas (non-ischemic areas and area at risk) stained brick red (red arrow) and the infarct was pale white (blue arrow), while Evans blue and TTC staining (**b**) determined the infarct area (white, blue arrow), area at risk (red, yellow arrow), and non-ischemic area (blue, red arrow).

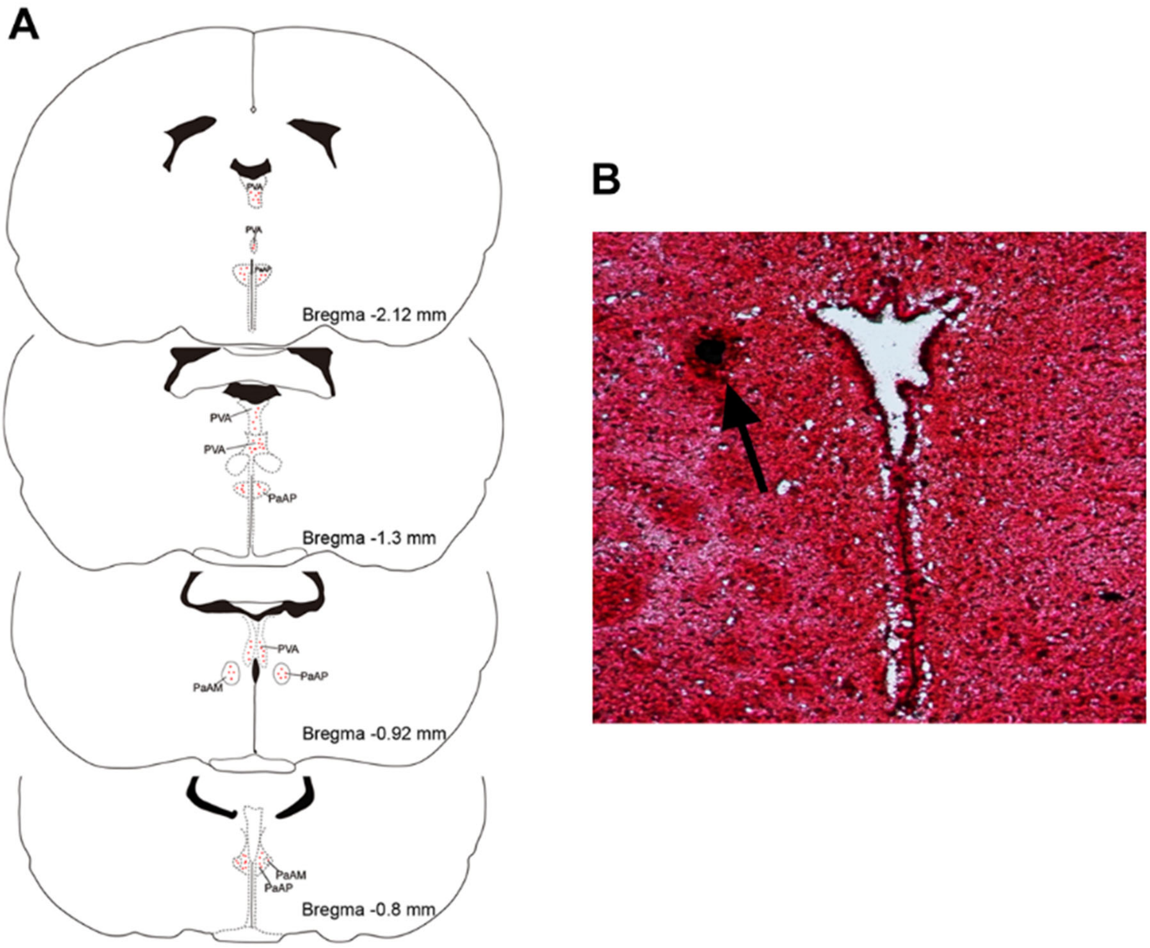

Fig S2 Histological identification of microinjection and recording sites. **A** Distributions of the microinjection and recording sites plotted on standard coronal sections according to the atlas of Paxinos and Watson [26] (red dots, microinjection sites for the PVN). **B** The black arrow indicates the microinjection site within the PVN.

Table S1 Effects of BBG or DPI pretreatment on HR and MAP of AMI rats

Groups	HR (beats/min)	MAP (mmHg)	*n*
Sham + Veh	430±1	117.6±1.2	8
AMI + Veh	504±4**	68.3±0.3**	8
BBG + AMI	454±4$^{\#\#}$	92.5±1.6$^{\#\#}$	6
DPI + AMI	443±24$^{\#\#}$	96.4±1.7$^{\#\#}$	6

Basal blood pressure and heart rate before treatment in all rats. Compared with the sham group, AMI rats showed a significantly lower MAP and higher HR, while the marked changes induced by infarction was slightly attenuated by administration of BBG or DPI. HR, heart rate; MAP, mean arterial blood pressure; BBG, brilliant blue G (a P2X7R antagonist); DPI, NADPH oxidase inhibitor; Veh, vehicle; aCSF, artificial cerebrospinal fluid; Vehicle: aCSF. Data are expressed as the mean ± SEM. $^{**}P < 0.01$ *vs* Sham group, $^{\#\#}P < 0.01$ *vs* AMI group.
